# Role of Sialidase in Long-Term Potentiation at Mossy Fiber-CA3 Synapses and Hippocampus-Dependent Spatial Memory

**DOI:** 10.1371/journal.pone.0165257

**Published:** 2016-10-26

**Authors:** Akira Minami, Masakazu Saito, Shou Mamada, Daisuke Ieno, Tomoya Hikita, Tadanobu Takahashi, Tadamune Otsubo, Kiyoshi Ikeda, Takashi Suzuki

**Affiliations:** 1 Department of Biochemistry, School of Pharmaceutical Sciences, University of Shizuoka, 52–1 Yada, Suruga-ku, Shizuoka 422–8526, Japan; 2 Department of Organic Chemistry, School of Pharmaceutical Sciences, Hiroshima International University, 5-1-1, Hirokoshingai, Kure-shi, Hiroshima 737–0112, Japan; Tokai University, JAPAN

## Abstract

Sialic acid bound to glycans in glycolipids and glycoproteins is essential for synaptic plasticity and memory. Sialidase (EC 3.2.1.18), which has 4 isozymes including Neu1, Neu2, Neu3 and Neu4, regulates the sialylation level of glycans by removing sialic acid from sialylglycoconjugate. In the present study, we investigated the distribution of sialidase activity in rat hippocampus and the role of sialidase in hippocampal memory processing. We previously developed a highly sensitive histochemical imaging probe for sialidase activity, BTP3-Neu5Ac. BTP3-Neu5Ac was cleaved efficiently by rat Neu2 and Neu4 at pH 7.3 and by Neu1 and Neu3 at pH 4.6. When a rat hippocampal acute slice was stained with BTP3-Neu5Ac at pH 7.3, mossy fiber terminal fields showed relatively intense sialidase activity. Thus, the role of sialidase in the synaptic plasticity was investigated at mossy fiber terminal fields. The long-term potentiation (LTP) at mossy fiber-CA3 pyramidal cell synapses was impaired by 2,3-dehydro-2-deoxy*-N*-acetylneuraminic acid (DANA), a sialidase inhibitor. DANA also failed to decrease paired-pulse facilitation after LTP induction. We also investigated the role of sialidase in hippocampus-dependent spatial memory by using the Morris water maze. The escape latency time to reach the platform was prolonged by DANA injection into the hippocampal CA3 region or by knockdown of Neu4 without affecting motility. The results show that the regulation of sialyl signaling by Neu4 is involved in hippocampal memory processing.

## Introduction

Sialic acid, an acidic monosaccharide, is expressed most frequently at the ends of glycans and creates a negative charge on the cell surface. Many neural functions including memory processing depend on sialic acid in glycoproteins and gangliosides [1–3]. Sialic acid contained in gangliosides such as the tetra-sialoganglioside GQ1b is crucial for synaptic plasticity and hippocampal memory [4, 5]. The sialic acid polymer (poly sialic acid, PSA), having a large negative charge, attached to neural cell adhesion molecules (NCAM) regulates neural circuit formation in memory and brain development [6, 7].

The sialic acid residue in a sialylglycoconjugate is removed by sialidase, which is one of the regulators for the sialylation level of glycans [8]. Since exogenous sialidase extracellularly applied to the hippocampus influences memory and synaptic plasticity [9, 10], endogenous sialidase activity in the extracellular space would also affect the hippocampal memory processing. In the case of inflammatory response in the central nervous system, sialylation level with PSA on microglial cells was regulated by exovesicular sialidase [11]. Mammalian sialidase isozymes are designated as Neu1, Neu2, Neu3 and Neu4. All sialidase isozymes, the main subcellular locations of which are different, show enzyme activity on the plasma membrane or in the extracellular space [8, 11, 12].

**Fig 1:**
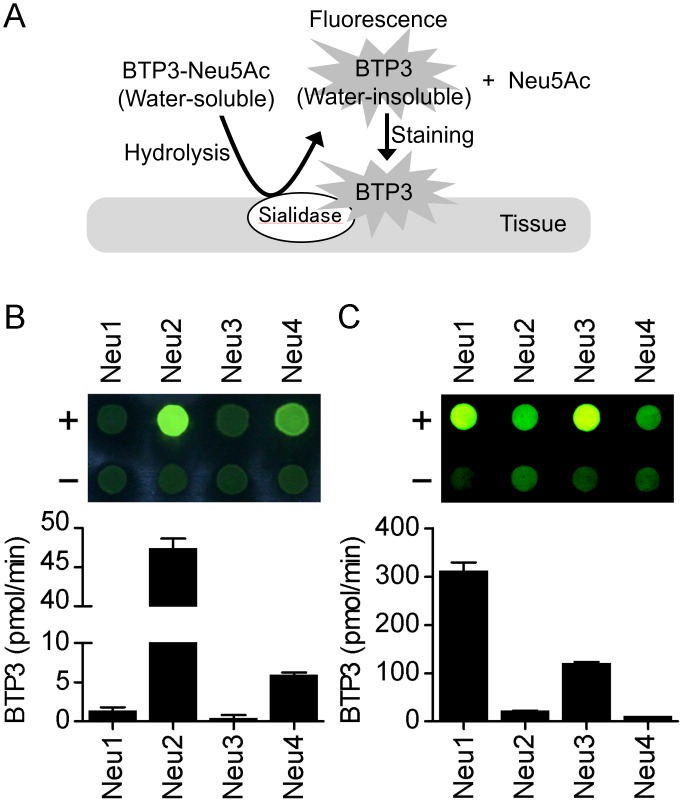
Difference in BTP3-Neu5Ac cleavage ability among sialidase isozymes. (A) Schematic staining mechanism of BTP3-Neu5Ac. (B and C) C-terminal Myc-tagged rat sialidase isozymes, Neu1, Neu2, Neu3 and Neu4, were expressed in C6 rat glioma cells. Lysate of sialidase isozyme-transfected cells (+) or mock-transfected cells (-, background level) was incubated in ACSF (pH 7.3, *n* = 4) (B) or sodium acetate buffer (pH 4.6, *n* = 4) (C) containing 200 μM BTP3-Neu5Ac. Then, fluorescence (green) was observed on a PVDF membrane under UV light (upper images). The amount of hydroryzed-BTP3 is shown as a bar graph after subtraction of each background level.

We developed a highly sensitive fluorescent histochemical imaging probe, benzothiazolylphenol-based sialic acid derivative (BTP3-Neu5Ac). When BTP3-Neu5Ac, which is water-soluble and shows little fluorescence, is cleaved by sialidase, the water-insoluble BTP3 having intense fluorescence under UV light is released and stains tissue clearly (Fig 1A) [13]. We also developed a fluorescent histochemical imaging method for sialidase activity by using the combination of 5-bromo-4-chloroindol-3-yl-α-D-N-acetylneuraminic acid (X-Neu5Ac) and Fast Red Violet LB (FRV LB) [14]. To visualize the distribution of extracellular sialidase activity, rat brain slices were stained with BTP3-Neu5Ac or X-Neu5Ac and FRV LB at neutral pH. The hippocampus showed weak but positive sialidase activity, although the white matter region showed the most intense sialidase activity in the brain [13, 14].

In the present study, we investigated the role of sialidase in hippocampal functions including synaptic plasticity and hippocampus-dependent spatial memory. We first determined the distribution of sialidase activity in the rat hippocampus by using BTP3-Neu5Ac. BTP3-Neu5Ac was cleaved efficiently by rat Neu2 and Neu4 at pH 7.3. As a result of rat hippocampal slice imaging with BTP3-Neu5Ac at neutral pH, mossy fiber terminals showed relatively intense sialidase activity. Thus, we next investigated the effects of a sialidase inhibitor on long-term potentiation (LTP) at mossy fiber-CA3 pyramidal cell synapses and hippocampus-dependent spatial memory. We also investigated the effect of Neu4 knockdown on hippocampus-dependent spatial memory.

## Materials and Methods

### Experimental animals

Male Wistar rats (3 weeks old for electrophysiological experiments and 8–9 weeks old for other experiments) were purchased from Japan SLC (Shizuoka, Japan). The rats were housed under standard laboratory conditions (23°C ± 1°C, 55% ± 5% humidity) and had access to tap water and diet *ad libitum*. The lights were automatically turned on at 8:00 and off at 20:00. The rats were handled for at least 10 min everyday for at least 7 days before use in the behavioral experiments. All experiments were performed in accordance with the guidelines established by the University of Shizuoka for the care and use of laboratory animals. The protocols were pre-approved by the Animal Ethics Committee of the University of Shizuoka. Euthanasia was performed under isoflurane anesthesia. All efforts were made to minimize suffering.

### Isolation and expression of each sialidase isozyme

Total mRNA of the rat liver (for Neu1, Neu2 and Neu3) or hippocampus (for Neu4) was prepared from a rat by using an RNeasy^®^ Plus Mini Kit (Qiagen, KJ Venlo, Netherlands) and converted to cDNA using a PrimeScript II High Fidelity One Step RT-PCR Kit (TaKaRa Bio, Shiga, Japan). The Neu1, Neu2, Neu3 and Neu4 genes were amplified using primers with restriction sites for ClaI (5'-CCATCGATATGGTGGGGGCAGAGCCAAG-3', 5'-CCATCGATATGGAGACCTGCCCCGTC-3', 5'-CCATCGATATGGAAGAAGTTTCATCCTGCTCCC-3' and 5'-CCATCGATATGGGGCCCGCGCATG-3', respectively) and MluI (5'-CGACGCGTGAGCGTGCCGTAGACGCTG-3', 5'-CGACGCGTCTGAGCACCATGTACTGTGGG-3', 5'-CGACGCGTGTTGCTACTAGGGCTGGTACAG-3' and 5'-CGACGCGTAGAGGGCCAGCAATGCCC-3', respectively) and PFU Ultra high-fidelity DNA polymerase (Agilent Technologies, Santa Clara, CA). The PCR products were digested using ClaI and MluI enzymes and then inserted into a multicloning site of the pQBI25-fPA vector (Wako Chemicals, Osaka, Japan). The nucleotide sequences of the sialidase isozyme genes in all plasmids were confirmed using an ABI Prism 310NT Genetic Analyzer (Applied Biosystems). Using these vectors as a PCR template, each sialidase isozyme gene was subcloned into the EcoRI and MluI restriction sites of the C-terminal myc tag-modified pBabe-puro retrovirus vector. Amplification of the Neu1, Neu2, Neu3 and Neu4 genes was performed using primers with the EcoRI (5’-CGGAATTCCCACCATGGTGGGGGCAGAGCC-3', 5’-CGGAATTCCCACCATGGAGACCTGCCCCGT-3’, 5’-CGGAATTCCCACCATGGAAGAAGTTTCATC-3’ and 5’-CGGAATTCCCACCATGGGGCCCGCGCATGTT-3’, respectively) and MluI (5’-CCGACGCGTGAGCGTGCCGTAGACGCT-3’, 5’-CCGACGCGTCTGAGCACCATGTACTGTG-3’, 5’-CCGACGCGTGTTGCTACTAGGGCTGGTA-3’ and 5’-CCGACGCGTAGAGGGCCAGCAATGCCC-3’, respectively) restriction sites and KOD plus DNA polymerase (Toyobo, Osaka, Japan). Each subcloned vector was transfected into plat-E cells with pGP + pE-Ampho (Takara Bio) using Lipofectamine 2000 (Invitrogen) and then the supernatants were infected into C6 rat glioma cells as retrovirus solution. Infected cell populations were selected using puromycin (4 μg/ml) for 1 week.

### Hydrolysis of BTP3-Neu5Ac with each sialidase isozyme

C6 rat glioma cells stably expressing one of the C-terminal Myc-tagged rat sialidase isozymes, Neu1, Neu2, Neu3 or Neu4, were lysed in n-octyl-β-D-glucoside (ODG) buffer [20 mM Tris-HCl (pH 7.4), 150 mM sodium chloride, 1 mM EDTA, 1 mM sodium orthovanadate, 20 mM sodium fluoride, 1% Nonidet P-40, 5% glycerol, 2% ODG, and a protease inhibitor cocktail (Nacalai Tesque)]. Each lysate-equalized amount of total protein was separated using sodium dodecyl sulphate polyacrylamide gel electrophoresis and transferred onto polyvinylidine difluoride (PVDF) membranes. The membranes were blocked and incubated using primary antibodies, followed by incubation with horseradish peroxidase (HRP)-conjugated secondary antibodies. The following primary antibodies were used: anti-myc-tag mAb (My3; MBL) and anti-beta actin (ab8226; Abcam). Signals from immune-positive bands were visualized using Lumiviewer (AISIN). Each lysate equalized with the amount of Myc were used to determine sialidase specificity to BTP3-Neu5Ac. For the background level measurement, the lysates of mock-transfected cells (negative control) that do not express myc were equalized with the amount of total protein in each sialidase isozyme.

For the hydrolysis of BTP3-Neu5Ac, each lysate was incubated in ACSF or 100 mM sodium acetate buffer (pH4.6) containing 200 μM BTP3-Neu5Ac at 27°C for 60 min. The released BTP3 was measured using a microplate reader (ex/em, 370 nm/526 nm) or observed using a digital camera under UV light (365 nm) after blotting on a PVDF membrane and a 96-well dot blotter (Sanplatec).

### Imaging of sialidase activity

The procedure for sialidase activity imaging was described previously [14]. Briefly, rat acute coronal brain slices prepared from 2 rats were incubated with 400 μl of artificial cerebrospinal fluid (ACSF, pH 7.3) containing 100 μM BTP3-Neu5Ac at 27°C for 60 min. The slices were washed with ice-cold ACSF and transferred to IWAKI 3.5-mm glass bottom dishes (Asahi Glass, Tokyo, Japan) filled with ACSF (27°C). Fluorescence was observed using a fluorescence microscope (IX71; Olympus) with a filter set (ex/em, BP330-385/BA510IF). The background level of fluorescence was determined using a non-stained brain slice. The gain of the DP70 Digital Microscope Camera (Olympus) was set in order to not detect background fluorescence. After acquiring the images, the images were ‘tiled’ together using Photoshop CS4. To confirm the specificity of imaging for sialidase activity, 2,3-dehydro-2-deoxy*-N*-acetylneuraminic acid (DANA) was applied during staining. Since it has been reported that IC_50_ values of DANA for Neu1, Neu2, Neu3 and Neu4 are 50–150 μM [15, 16], 10 mM of DANA was used for complete inhibition of mammalian sialidase activity. To keep the brain slices healthy, all solutions used in the acute slice experiments were continuously bubbled with 95% O2 and 5% CO2. Staining was repeated 4 times and reproducibility was confirmed.

### Electrophysiology

The mossy fiber-CA3 LTP protocol followed the method reported by Honda *et al*. [17]. An acute hippocampal slice prepared from 7 rats was transferred to the recording chamber under perfusion with ACSF (1 ml/min, 25–26°C) or ACSF containing 300 μM DANA (Tokyo Chemical Industry, Tokyo, Japan). Electrical stimuli were delivered through a tungsten bipolar electrode inserted into the stratum granulosum of the dentate gyrus at 0.05 Hz unless otherwise stated. Field excitatory postsynaptic potentials (fEPSPs) were recorded from the CA3 stratum lucidum using a glass electrode (3 M NaCl, 1–2 MΩ) through low-pass (1 kHz) and high-pass (1 Hz) filters. Tetanic stimulation (25 Hz, 5 s) was applied in the presence of 50 μM 2-amino-5-phosphonopentanoic acid (AP5). After LTP recording, 1 μM (2S,2'R,3'R)-2-(2',3'-dicarboxycyclopropyl)glycine (DCG-IV) was applied. In the case of failure to reduce fEPSP to less than 10% with DCG-IV, the data were not used. At the end of each experiment, 10 μM 6-cyano-7-nitroquinoxaline-2,3-dione (CNQX) was applied. Paired-pulse facilitation (PPF) was induced by stimulating twice with a 40-ms interval.

### DANA injection into the hippocampus

DANA injection into the hippocampus was performed using a method similar to that previously described [18]. Briefly, guide cannulae with dummy injection cannulae were surgically implanted into the bilateral hippocampi (AP = −5.6 mm; ML = ±4.6 mm; DV = 5.1 mm) using a stereotaxic instrument (Narishige, Tokyo, Japan) [19] and fixed using dental cement under chloral hydrate anaesthesia (400 mg/kg body weight). After surgery, animal condition was monitored carefully especially during recovery term from anesthesia. Three days after implantation, dummy injection cannulae were replaced with injection cannulae. Five μl of ACSF or ACSF containing 5 mM DANA was injected into the bilateral hippocampi through the injection cannulae at 0.1 μl/s in the awakened state. After standing still for 3 minutes, the injection cannulae were replaced with dummy injection cannulae immediately. Injection was performed once a day at 10 min before the first trial in the Morris water maze test. After the behavioral experiments, 5 μl of 1% Evans blue was injected in the same manner to confirm the diffusion and injection region.

### Morris water maze test

The Morris water maze test was performed as previously described [20]. Briefly, a transparent platform (10 cm in diameter) was submerged in a pool (130 cm in diameter) filled with water (22°C ± 2°C) and surrounded by a grey wall (57 cm in height) and two different objects. Rats were released into the water facing the wall and trained to find the platform for a maximum of 40 s. In the case of failures, the rats were guided to the platform by hand. After reaching the platform, the rats were kept on the platform for 10 s and then picked up. Each trial was repeated four times with 1-min intervals, which comprised 1 set. The releasing location was changed in each trial. The rats were trained for two sets a day up to 2 or 3 days (training session). Daily training was started at a fixed time, and a second set was performed 3 h after the first set. After the training session, the rats were allowed to swim for 60 s in the absence of the platform. Memory for the platform location was assessed by quantifying the time spent in the quadrant in which the platform had been previously placed in the training session (probe test). For motility assessment, the total number of times a rat entered each quadrant was counted in the first training of the first trial on day 1. The experiment was performed in a double-blind manner.

### Knockdown by siRNA

An L-shaped cannula (Durect, CA, USA) was surgically implanted into the dorsal third ventricle (AP = −4.2 mm; ML = 0.0 mm; DV = 4.6 mm). Double-stranded small interfering RNAs (siRNAs) targeting rat Neu4 (GAGACUUUCUCUACUGUAATT) or a non-targeting double-stranded siRNA (UGGUUUACAUGUCGACUAATT) were obtained from Hokkaido System Science (Hokkaido, Japan). These siRNAs were diluted with an *in vivo* siRNA transfection reagent (AteloGene^®^; Koken, Tokyo, Japan) and continuously injected through the cannula for 7 days using an Alzet mini-osmotic pump^®^ (Durect) implanted in the dorsal subcutaneous tissue. Behavioral experiments were performed 3 days after the start of injection. To minimize the off-target effect, siRNA sequences for Neu4 knockdown were chosen using siDirect version 2.0.

### Real-time quantitative reverse transcription-polymerase chain reaction (real-time RT-PCR)

The procedure for real-time RT-PCR was in accordance with a method reported previously [21]. Total RNA was purified from brain tissues using an RNeasy^®^ Plus Mini Kit. *Neu4* cDNA copies were evaluated using RT-PCR (LightCycler 2.0; Roche Diagnostics, Basel, Switzerland), a One Step SYBR PrimeScript PLUS RT-PCR kit (Perfect Real Time, TaKaRa Bio) and primer pairs [5′-TCTGGAGAGTGCCAACTGGC-3′ and 5′-AAGGAAGTGCCTTCATCAGCAC-3′ for Neu4; 5′-TGAACGGATTTGGCCGTATCGG-3′ and 5′-TCAATGAAGGGGTCGTTGATGG-3′ for glyceraldehyde-3-phosphate dehydrogenase (GAPDH)]. Standard curves of Neu4 or GAPDH cDNA copies (cycle values vs. cDNA copies) were constructed using data obtained by serial dilution of total RNA obtained from the rat hippocampus injected with non-targeting siRNA. To normalize for sample variation, cDNA copies of GAPDH were determined as an internal control.

### Statistical analysis

Statistical significance was assessed using two-tailed unpaired t-test with Welch's correction, two-tailed paired t-test, one-way ANOVA with Dunnett's multiple comparison test, Kruskal-Wallis test, and one-way or two-way repeated measures ANOVA with Bonferroni's multiple comparison test. Statistical analysis was performed using Prism 5 (GraphPad, La Jolla, CA). Error bars are expressed as standard errors of the mean.

## Results

### Cleavage of BTP3-Neu5Ac with rat sialidase isozymes

The staining mechanism of BTP3-Neu5Ac is schematically shown in Fig 1A. Briefly, BTP3-Neu5Ac is water-soluble and has little fluorescence. When BTP3-Neu5Ac is hydrolyzed with sialidase, BTP3 shows intense fluorescence. Since BTP3 is a water-insoluble fluorophore, tissue is stained with BTP3. To compare the cleavage abilities of BTP3-Neu5Ac among recombinant rat sialidase isozymes, we constructed C-terminal Myc-tagged rat sialidase isozymes in C6 glioma cells. BTP3-Neu5Ac was hydrolyzed preferentially by Neu2 and Neu4 and weakly by Neu1 and Neu3 at pH 7.3 (Fig 1B). At pH 4.6, BTP3-Neu5Ac was hydrolyzed efficiently by Neu1 and Neu3 and also by Neu2 and Neu4 (Fig 1C).

### Imaging of sialidase activity in rat hippocampus with BTP3-Neu5Ac

**Fig 2:**
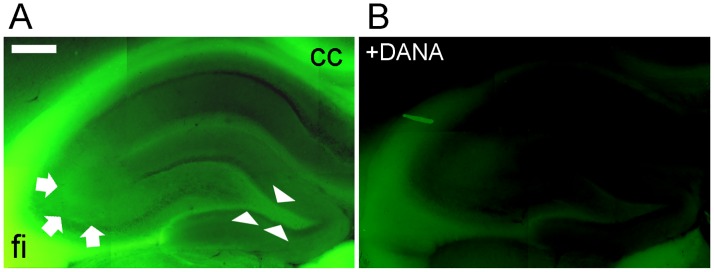
Sialidase activity imaging in rat hippocampus. Rat hippocampus and the surrounding region was stained with 100 μM BTP3-Neu5Ac (A) or with 100 μM BTP3-Neu5Ac +10 mM DANA (B) at pH 7.3. cc: corpus callosum, fi: hippocampal fimbria. Arrows, CA3 striatum lucidum; arrowheads, hilus. Scale bar, 0.5 mm.

We investigated the distribution of sialidase activity in the rat hippocampus by using BTP3-Neu5Ac. When acute brain slices including the hippocampus were stained with BTP3-Neu5Ac at pH 7.3, the white matter regions including corpus callosum and hippocampal fimbria showed intense fluorescence. In the hippocampus, the CA3 stratum lucidum and hilus of the dentate gyrus, where mossy fibre terminate, showed relatively intense fluorescence (Fig 2A). Sialidase inhibitor, a 2,3-dehydro-2-deoxy*-N*-acetylneuraminic acid (DANA), remarkably attenuated the fluorescence caused by staining with BTP3-Neu5Ac, indicating that BTP3-Neu5Ac specifically detected sialidase activity (Fig 2B).

### Effect of sialidase inhibitor on LTP at the mossy fiber-CA3 synapses

**Fig 3:**
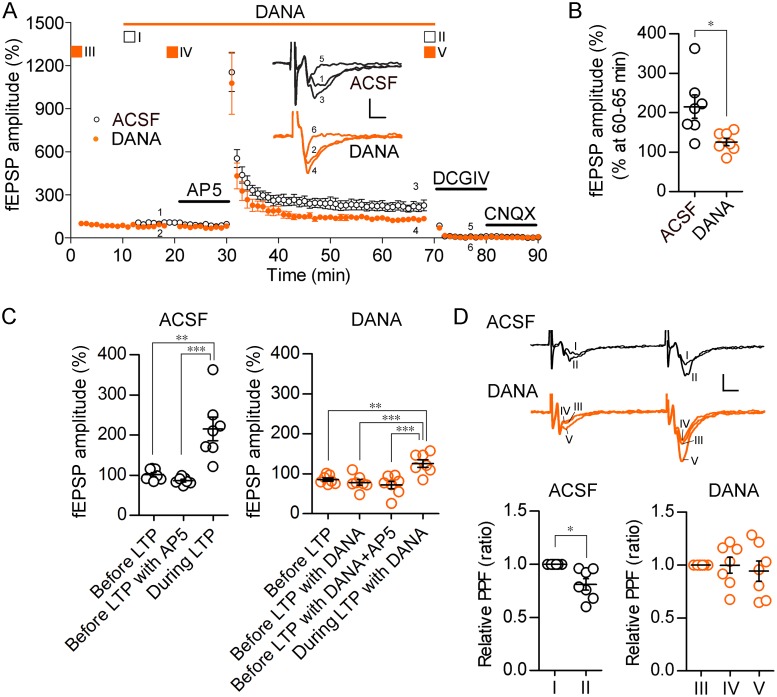
Impairment of mossy fiber-CA3 LTP by a sialidase inhibitor. (A) LTP was induced at 30 min by tetanic stimulation in the presence of 50 μM AP5. DANA (300 μM), 1 μM DCG-IV and 10 μM CNQX were applied at 10–70, 70–80 and 80–90 min, respectively. *n* = 7 each. The inset shows representative fEPSPs recorded at the times indicated by numbers in the graph. Scale bars, 0.1 mV and 5 ms. (B) The magnitudes of LTP were averaged at 60–65 min. *P < 0.05 (unpaired *t*-test with Welch's correction). (C) fEPSPs were averaged at 13–18 min (before LTP), 25–30 min (before LTP with AP5) and 60–65 min (during LTP) in the vehicle (ACSF)-treated group and at 5–10 min (before LTP), 13–18 min (before LTP with DANA), 25–30 min (before LTP with DANA+AP5) and 60–65 min (during LTP with DANA) in the DANA-treated group. **P < 0.01, ***P < 0.001 (repeated measures ANOVA with Bonferroni's multiple comparison test) (D) PPF was measured at the times indicated by squares (white: ACSF, orange: DANA) and roman numerals in panel (A). *P < 0.05 (paired *t*-test).

Based on the finding that relative intense sialidase activity was observed in mossy fiber terminal fields, we focused on the function of sialidase in LTP at the mossy fiber-CA3 synapses. LTP at the mossy fiber-CA3 synapses can be induced by an *N*-methyl-*D*-aspartate receptor-independent (NMDAR-independent) presynaptic pathway [22] (but see about an NMDAR-mediated pathway [23, 24]). The NMDAR-independent LTP induced by tetanic stimulation (25 Hz, 5 s) in the presence of AP5, an NMDAR antagonist, was attenuated by DANA (Fig 3A and 3B). After LTP recording, DCG-IV, a group II metabotropic glutamate receptor agonist, was applied to confirm that the recorded fEPSP originated from the mossy fiber-CA3 synapses. At the end of each experiment, CNQX, an α-amino-3-hydroxy-5-methyl-4-isoxazolepropionic acid (AMPA)/kainate receptor antagonist, was applied to isolate the fEPSP from the presynaptic fiber volley component (Fig 3A). DANA did not affect the basal fEPSP levels (Fig 3C).

### Effect of sialidase inhibitor on PPF reduction after LTP induction

PPF is induced by two stimuli delivered at a 40-ms interval. The Ca^2+^ transient elicited by the first stimulation in presynaptic terminals results in the enhancement of postsynaptic potentiation in response to the second stimulation. Thus, PPF is a presynaptic parameter associated with neurotransmitter release probability. Because NMDAR-independent LTP at the mossy fiber-CA3 synapses is expressed by long-lasting enhancement of transmitter release, PPF evoked by stimulating the mossy fiber twice at a 40-ms interval was decreased after LTP induction [25, 26]. However, decrease in PPF after LTP induction was failed in the presence of DANA (one-way ANOVA, *F*_*2*,*18*_ = 0.21) (Fig 3D).

### Roles of sialidase in hippocampal spatial memory

**Fig 4:**
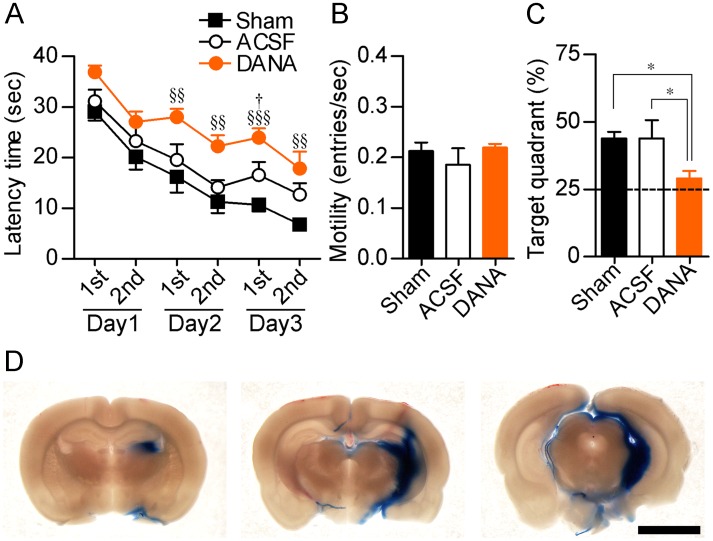
Impairment of hippocampus-dependent memory by a sialidase inhibitor. (A) A Morris water maze test was performed after injection of 5 μl of ACSF or 5 mM DANA into the rat bilateral hippocampi. Latency time until reaching the hidden platform was measured (training session). Sham, *n* = 12; ACSF, *n* = 7; DANA, *n* = 7. ^§§^*P* < 0.01 and ^§§§^*P* < 0.001 vs. Sham; ^†^*P* < 0.05 vs. ACSF; two-way repeated measures ANOVA with Bonferroni's multiple comparison test. (B) Motility was assessed in the first trial of the training session on day 1. (C) A probe test was performed after the training session. Dashed lines represent the chance level. *P < 0.05 (one-way ANOVA with Dunnett's Multiple Comparison Test, *F*_*2*,*22*_ = 4.83). (D) Diffusion pattern after injection of 5 μl of 1% Evans blue in ACSF into the right hippocampus (AP = −5.6 mm; ML = +4.6 mm; DV = 5.1 mm). Scale bar, 5 mm.

We next directly assessed the role of sialidase in hippocampus-dependent spatial memory. In a training session of the Morris water maze test, the latency time to reach the platform was significantly prolonged by injection of 5 mM DANA (5 μl) into the rat bilateral hippocampi (Fig 4A). The motility was not affected by injection of DANA (Kruskal-Wallis test) (Fig 4B). In the probe test session, quadrant occupancy measured by quantifying the time spent in the target quadrant was reduced by DANA injection (Fig 4C). To estimate the diffusion region of DANA, 1% Evans blue dye (5 μl) was injected in the same manner. Most part of the dye was remained in the hippocampal dentate gyrus and CA3 regions 20 min after injection (Fig 4D).

### Importance of sialidase isozyme Neu4 in hippocampal spatial memory

**Fig 5:**
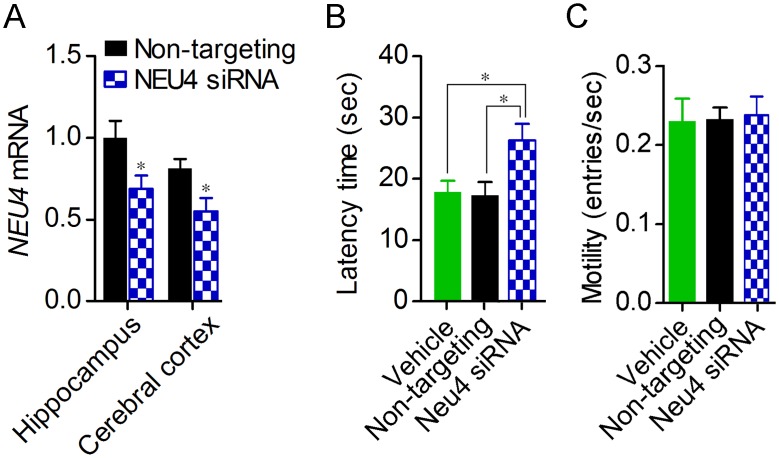
Impairment of hippocampus-dependent memory by knockdown of Neu4. (A) *Neu4* mRNA levels in the hippocampus and cerebral cortex were measured after continuous injection of Neu4-targeting (*n* = 9) or non-targeting siRNA (*n* = 10) into the dorsal third ventricle for 7 days. *P < 0.05 vs. non-targeting siRNA (unpaired t*-test*). (B) Morris water maze test was performed under the effect of Neu4-targeting siRNA (*n* = 8), non-targeting (*n* = 10) or vehicle (*n* = 10). Latency time at the 1^st^ trial on day 2 was statistically analysed (one-way ANOVA with Newman-Keuls Multiple Comparison Test, *F*_2,25_ = 4.77). (C) Motility was assessed in the first trial of the training session on day 1.

We investigated the role of the sialidase isozyme Neu4 in hippocampal spatial memory. Since DANA impaired memory after the first trial on day 2, the effect of Neu4 knockdown on hippocampal spatial memory was examined at the first trial on day 2. Continuous delivery of siRNA targeting Neu4 into the rat cerebral ventricle for 7 days significantly decreased *Neu4* mRNA levels in the hippocampus by 31.2% and in the cerebral cortex by 32.1% (Fig 5A). As a result of weak Neu4 knockdown, hippocampal memory was impaired in the first trial on day 2 (Fig 5B). Motility was not affected by Neu4 knockdown (one-way ANOVA, *F*_*2*,*26*_ = 0.03) (Fig 5C).

## Discussion

We first compared the cleavage abilities of BTP3-Neu5Ac among recombinant rat sialidase isozymes. BTP3-Neu5Ac was hydrolyzed preferentially by Neu2 and Neu4 in ACSF (pH 7.3). Neu4 mRNA is expressed predominantly in the brain, particularly in the hippocampus [27, 28]. Neu4 has broad pH dependency with optimal pH of 3.5–4.6 and shows sialidase activity even at neutral pH [29, 30]. In contrast, Neu2 is poorly expressed in the brain [31, 32]. Thus, Neu4 would mainly contribute to the sialidase activity detected by BTP3-Neu5Ac at pH 7.3 in the brain.

To visualize the distribution of extracellular sialidase activity in hippocampus, we previously stained the rat brain slice with X-Neu5Ac and FRV LB at pH 7.3 [14]. Although the hippocampus showed weak fluorescence compared to the white matter, mossy fiber terminal fields showed relatively intense fluorescence in the hippocampus. In the present study, the rat hippocampus was stained with BTP3-Neu5Ac at pH 7.3. Consistent with our previous results, mossy fiber terminal fields including the CA3 stratum lucidum and hilus of the dentate gyrus showed intense sialidase activity in the hippocampus. Sialidase activity would be caused mainly by mossy fiber terminals, but other cells such as glia cells may also contribute to the sialidase activity and regulation of neural functions. Although sialidase, especially Neu4, is estimated to contribute the function at mossy fiber-CA3 synapses, role of sialidase in hippocampus has been poorly understood.

The importance of sialic acid in the synaptic plasticity has been reported in hippocampal CA1 regions. At Schaffer collateral-CA1 synapses, the tetra-sialoganglioside GQ1b increased the magnitude of LTP much more than did the mono-sialoganglioside GM1 [4, 33–35]. Removal of PSA with endo-N, an endosialidase that specifically cleaves α2,8-linked sialic acid, causes impairment of long-term depression as well as LTP. The mutant mice lacking ST8SiaIV, a sialyltransferase for PSA production, exhibit abnormal LTP at Schaffer collateral-CA1 synapses [36]. Since inhibition of sialidase impairs LTP at Schaffer collateral-CA1 synapses, regulation of sialyl signaling by sialidase is necessary for memory processing in the CA1 region [37].

The mechanism by which LTP is induced at mossy fiber-CA3 synapses is different from that by which LTP is induced at Schaffer collateral-CA1 synapses. LTP at Schaffer collateral-CA1 synapses is postsynaptically induced by activation of NMDAR. On the other hand, LTP at mossy fiber-CA3 synapses is presynaptically induced without activation of NMDAR [22]. Here, we investigated the role of intrinsic sialidase in LTP at mossy fiber-CA3 synapses. Chemical inhibition of sialidase activity by DANA impaired LTP at mossy fiber-CA3 synapses. Decrease in PPF after LTP induction was also impaired by DANA. Thus, sialidase is necessary for enhancement of transmitter release in the LTP at mossy fiber-CA3 synapses.

The hippocampus-dependent spatial memory process is related to not only CA1 activity but also CA3 activity [38–40] (but see [41]). Here, we investigated the role of sialidase in hippocampus-dependent spatial memory by using the Morris water maze. The escape latency was prolonged by DANA injection into the hippocampal CA3 region or by knockdown of Neu4 without affecting motility. Although DANA has an inhibitory effect on all mammalian sialidase isozymes [15], at least Neu4 seems to be involved in hippocampus-dependent memory. However, whether Neu4 knockdown can inhibit LTP at mossy fiber-CA3 synapses remains an open question, and further experiments are required.

In addition to lysosomes, mitochondria and endoplasmic reticulum, Neu4 is localized at the cell surface [8, 12, 30, 42]. Since sialidase activity in the mossy fiber terminal region was detected at neutral pH, a part of Neu4 would work on the cell surface. Desialylation on the cell surface by sialidase can change cell surface properties quickly and dramatically. Neu1 is also contributed to the desialylation on the cell surface in the central nervous system [11]. Neu3, plasma-membrane-bound sialidase, cleaves sialic acid bound to the major brain gangliosides that are necessary for memory function. Thus, there is a possibility that sialidase isozymes other than Neu4 also contribute to hippocampal spatial memory.

Neu4 catalyzes removal of sialic acid from glycoproteins, gangliosides and oligosaccharides as well as degradation of PSA [27, 28, 42]. PSA regulates the activity of voltage-gated sodium channels and glutamate receptors [43, 44] and has capturing ability of BDNF and dopamine [45, 46]. PSA also plays an important role in hippocampal synaptic plasticity and memory [6]. The expression level of PSA on NCAM in hippocampal mossy fiber terminals is related to synaptic maturation. PSA on NCAM was negative in most of the mature mossy fiber boutons but was positive in immature boutons. PSA-positive boutons form invagination from finger-like dendritic outgrowth of pyramidal cells, which develop into a thorny excrescence [47]. These findings support the idea that PSA removal by Neu4 induces synaptic maturation at mossy fiber boutons in memory processing.

## Abbreviations

ACSF: artificial cerebrospinal fluid
AMPA: α-amino-3-hydroxy-5-methyl-4-isoxazolepropionic acid
AP5: 2-amino-5-phosphonopentanoic acid
BTP3-Neu5Ac: benzothiazolylphenol-based sialic acid derivative
CNQX: 6-cyano-7-nitroquinoxaline-2,3-dione
DANA: 2,3-dehydro-2-deoxy*-N*-acetylneuraminic acid
DCG-IV: (2S,2'R,3'R)-2-(2',3'-dicarboxycyclopropyl)glycine
fEPSP: field excitatory postsynaptic potential
LTP: long-term potentiation
NCAM: neural cell adhesion molecules
NMDAR: *N*-methyl-*D*-aspartate receptor
PPF: paired-pulse facilitation
real-time RT-PCR: real-time quantitative reverse transcription-polymerase chain reaction

## References

[1] Sato C, Kitajima K (2013) Disialic, oligosialic and polysialic acids: distribution, functions and related disease. J Biochem 154(2):115–36. 10.1093/jb/mvt057 23788662

[2] Schnaar RL, Gerardy-Schahn R, Hildebrandt H (2014) Sialic acids in the brain: gangliosides and polysialic acid in nervous system development, stability, disease, and regeneration. Physiol Rev 94(2):461–518. 10.1152/physrev.00033.2013 24692354 PMC4044301

[3] Minami A, Suzuki T (2012) Distribution of sialidase activity and the role of sialidase in the brain. Trends in Glycosci Glycotech 24(137):112–21.

[4] Fujii S, Igarashi K, Sasaki H, Furuse H, Ito K, Kaneko K, et al (2002) Effects of the mono- and tetrasialogangliosides GM1 and GQ1b on ATP-induced long-term potentiation in hippocampal CA1 neurons. Glycobiology 12(5):339–44. 12070076 10.1093/glycob/12.5.339

[5] Jung WR, Kim HG, Kim KL (2008) Ganglioside GQ1b improves spatial learning and memory of rats as measured by the Y-maze and the Morris water maze tests. Neurosci Lett 439(2):220–5. 10.1016/j.neulet.2008.05.020 18514410

[6] Gascon E, Vutskits L, Kiss JZ (2007) Polysialic acid-neural cell adhesion molecule in brain plasticity: from synapses to integration of new neurons. Brain Res Rev 56(1):101–18. 10.1016/j.brainresrev.2007.05.014 17658613

[7] Senkov O, Tikhobrazova O, Dityatev A (2012) PSA-NCAM: synaptic functions mediated by its interactions with proteoglycans and glutamate receptors. Int J Biochem Cell Biol 44(4):591–5. 10.1016/j.biocel.2012.01.008 22300986

[8] Miyagi T, Yamaguchi K (2012) Mammalian sialidases: physiological and pathological roles in cellular functions. Glycobiology 22(7):880–96. 10.1093/glycob/cws057 22377912

[9] Kochlamazashvili G, Senkov O, Grebenyuk S, Robinson C, Xiao MF, Stummeyer K, et al (2010) Neural cell adhesion molecule-associated polysialic acid regulates synaptic plasticity and learning by restraining the signaling through GluN2B-containing NMDA receptors. J Neurosci 30(11):4171–83. 10.1523/JNEUROSCI.5806-09.2010 20237287 PMC5390116

[10] Muller D, Wang C, Skibo G, Toni N, Cremer H, Calaora V, et al (1996) PSA-NCAM is required for activity-induced synaptic plasticity. Neuron 17(3):413–22. 8816705 10.1016/s0896-6273(00)80174-9

[11] Sumida M, Hane M, Yabe U, Shimoda Y, Pearce OM, Kiso M, et al (2015) Rapid trimming of cell surface polySia by exovesicular sialidase triggers release of preexisting surface neurotrophin. J Biol Chem 290(21):13202–14. 10.1074/jbc.M115.638759 25750127 PMC4505574

[12] Shiozaki K, Yamaguchi K, Takahashi K, Moriya S, Miyagi T (2011) Regulation of sialyl Lewis antigen expression in colon cancer cells by sialidase NEU4. J Biol Chem 286(24):21052–61. 10.1074/jbc.M111.231191 21521691 PMC3122166

[13] Minami A, Otsubo T, Ieno D, Ikeda K, Kanazawa H, Shimizu K, et al (2014) Visualization of sialidase activity in Mammalian tissues and cancer detection with a novel fluorescent sialidase substrate. PLoS One 9(1):e81941 10.1371/journal.pone.0081941 24427265 PMC3888388

[14] Minami A, Shimizu H, Meguro Y, Shibata N, Kanazawa H, Ikeda K, et al (2011) Imaging of sialidase activity in rat brain sections by a highly sensitive fluorescent histochemical method. Neuroimage 58(1):34–40. 10.1016/j.neuroimage.2011.06.017 21703353

[15] Hata K, Koseki K, Yamaguchi K, Moriya S, Suzuki Y, Yingsakmongkon S, et al (2008) Limited inhibitory effects of oseltamivir and zanamivir on human sialidases. Antimicrob Agents Chemother 52(10):3484–91. 10.1128/AAC.00344-08 18694948 PMC2565904

[16] Magesh S, Moriya S, Suzuki T, Miyagi T, Ishida H, Kiso M (2008) Design, synthesis, and biological evaluation of human sialidase inhibitors. Part 1: selective inhibitors of lysosomal sialidase (NEU1). Bioorg Med Chem Lett 18(2):532–7. 10.1016/j.bmcl.2007.11.084 18068975

[17] Honda T, Sakisaka T, Yamada T, Kumazawa N, Hoshino T, Kajita M, et al (2006) Involvement of nectins in the formation of puncta adherentia junctions and the mossy fiber trajectory in the mouse hippocampus. Mol Cell Neurosci 31(2):315–25. 10.1016/j.mcn.2005.10.002 16300961

[18] Minami A, Takeda A, Yamaide R, Oku N (2002) Relationship between zinc and neurotransmitters released into the amygdalar extracellular space. Brain Res 936(1–2):91–4. 11988235 10.1016/s0006-8993(02)02499-x

[19] Paxinos G, Watson C. The rat brain in stereotaxic coordinates. 6 ed Amsterdam, Boston: Academic Press/Elsevier; 2007. 1 v. (unpaged) p.

[20] Minami A, Matsushita H, Horii Y, Ieno D, Matsuda Y, Saito M, et al (2013) Improvement of depression-like behavior and memory impairment with the ethanol extract of Pleurotus eryngii in ovariectomized rats. Biol Pharm Bull 36(12):1990–5. 24292057 10.1248/bpb.b13-00657

[21] Takahashi T, Kawakami T, Mizuno T, Minami A, Uchida Y, Saito T, et al (2013) Sensitive and direct detection of receptor binding specificity of highly pathogenic avian influenza A virus in clinical samples. PLoS One 8(10):e78125 10.1371/journal.pone.0078125 24205123 PMC3799784

[22] Nicoll RA, Malenka RC (1995) Contrasting properties of two forms of long-term potentiation in the hippocampus. Nature 377(6545):115–8. 10.1038/377115a0 7675078

[23] Kwon HB, Castillo PE (2008) Long-term potentiation selectively expressed by NMDA receptors at hippocampal mossy fiber synapses. Neuron 57(1):108–20. 10.1016/j.neuron.2007.11.024 18184568 PMC2390917

[24] Rebola N, Carta M, Lanore F, Blanchet C, Mulle C (2011) NMDA receptor-dependent metaplasticity at hippocampal mossy fiber synapses. Nat Neurosci 14(6):691–3. 10.1038/nn.2809 21532578

[25] Katafuchi T, Li AJ, Hirota S, Kitamura Y, Hori T (2000) Impairment of spatial learning and hippocampal synaptic potentiation in c-kit mutant rats. Learn Mem 7(6):383–92. 11112797 10.1101/lm.33900 PMC311355

[26] Zalutsky RA, Nicoll RA (1990) Comparison of two forms of long-term potentiation in single hippocampal neurons. Science 248(4963):1619–24. 2114039 10.1126/science.2114039

[27] Comelli EM, Amado M, Lustig SR, Paulson JC (2003) Identification and expression of Neu4, a novel murine sialidase. Gene 321:155–61. 14637003 10.1016/j.gene.2003.08.005

[28] Shiozaki K, Koseki K, Yamaguchi K, Shiozaki M, Narimatsu H, Miyagi T (2009) Developmental change of sialidase neu4 expression in murine brain and its involvement in the regulation of neuronal cell differentiation. J Biol Chem 284(32):21157–64. 10.1074/jbc.M109.012708 19506080 PMC2755838

[29] Seyrantepe V, Landry K, Trudel S, Hassan JA, Morales CR, Pshezhetsky AV (2004) Neu4, a novel human lysosomal lumen sialidase, confers normal phenotype to sialidosis and galactosialidosis cells. J Biol Chem 279(35):37021–9. 10.1074/jbc.M404531200 15213228

[30] Shiozaki K, Ryuzono S, Matsushita N, Ikeda A, Takeshita K, Chigwechokha PK, et al (2014) Molecular cloning and biochemical characterization of medaka (Oryzias latipes) lysosomal neu4 sialidase. Fish Physiol Biochem 40(5):1461–72. 10.1007/s10695-014-9940-9 24744226

[31] Hasegawa T, Feijoo Carnero C, Wada T, Itoyama Y, Miyagi T (2001) Differential expression of three sialidase genes in rat development. Biochem Biophys Res Commun 280(3):726–32. 10.1006/bbrc.2000.4186 11162581

[32] Monti E, Preti A, Rossi E, Ballabio A, Borsani G (1999) Cloning and characterization of NEU2, a human gene homologous to rodent soluble sialidases. Genomics 57(1):137–43. 10.1006/geno.1999.5749 10191093

[33] Furuse H, Waki H, Kaneko K, Fujii S, Miura M, Sasaki H, et al (1998) Effect of the mono- and tetra-sialogangliosides, GM1 and GQ1b, on long-term potentiation in the CA1 hippocampal neurons of the guinea pig. Exp Brain Res 123(3):307–14. 9860269 10.1007/s002210050573

[34] Ramirez OA, Gomez RA, Carrer HF (1990) Gangliosides improve synaptic transmission in dentate gyrus of hippocampal rat slices. Brain Res 506(2):291–3. 2302568 10.1016/0006-8993(90)91264-h

[35] Wieraszko A, Seifert W (1985) The role of monosialoganglioside GM1 in the synaptic plasticity: in vitro study on rat hippocampal slices. Brain Res 345(1):159–64. 2998547 10.1016/0006-8993(85)90847-9

[36] Eckhardt M, Bukalo O, Chazal G, Wang L, Goridis C, Schachner M, et al (2000) Mice deficient in the polysialyltransferase ST8SiaIV/PST-1 allow discrimination of the roles of neural cell adhesion molecule protein and polysialic acid in neural development and synaptic plasticity. J Neurosci 20(14):5234–44. 10884307 10.1523/JNEUROSCI.20-14-05234.2000 PMC6772332

[37] Savotchenko A, Romanov A, Isaev D, Maximyuk O, Sydorenko V, Holmes GL, et al (2015) Neuraminidase inhibition primes short-term depression and suppresses long-term potentiation of synaptic transmission in the rat hippocampus. Neural Plast 2015:908190 10.1155/2015/908190 25802763 PMC4329761

[38] Ramirez-Amaya V, Balderas I, Sandoval J, Escobar ML, Bermudez-Rattoni F (2001) Spatial long-term memory is related to mossy fiber synaptogenesis. J Neurosci 21(18):7340–8. 11549744 10.1523/JNEUROSCI.21-18-07340.2001 PMC6763009

[39] Meilandt WJ, Barea-Rodriguez E, Harvey SA, Martinez JL Jr. (2004) Role of hippocampal CA3 mu-opioid receptors in spatial learning and memory. J Neurosci 24(12):2953–62. 10.1523/JNEUROSCI.5569-03.2004 15044534 PMC6729851

[40] Gilbert PE, Brushfield AM (2009) The role of the CA3 hippocampal subregion in spatial memory: a process oriented behavioral assessment. Prog Neuropsychopharmacol Biol Psychiatry 33(5):774–81. 10.1016/j.pnpbp.2009.03.037 19375477 PMC2743458

[41] Hensbroek RA, Kamal A, Baars AM, Verhage M, Spruijt BM (2003) Spatial, contextual and working memory are not affected by the absence of mossy fiber long-term potentiation and depression. Behav Brain Res 138(2):215–23. 12527452 10.1016/s0166-4328(02)00243-7

[42] Takahashi K, Mitoma J, Hosono M, Shiozaki K, Sato C, Yamaguchi K, et al (2012) Sialidase NEU4 hydrolyzes polysialic acids of neural cell adhesion molecules and negatively regulates neurite formation by hippocampal neurons. J Biol Chem 287(18):14816–26. 10.1074/jbc.M111.324186 22393058 PMC3340223

[43] Hammond MS, Sims C, Parameshwaran K, Suppiramaniam V, Schachner M, Dityatev A (2006) Neural cell adhesion molecule-associated polysialic acid inhibits NR2B-containing N-methyl-D-aspartate receptors and prevents glutamate-induced cell death. J Biol Chem 281(46):34859–69. 10.1074/jbc.M602568200 16987814

[44] Hoffman KB, Kessler M, Lynch G (1997) Sialic acid residues indirectly modulate the binding properties of AMPA-type glutamate receptors. Brain Res 753(2):309–14. 9125416 10.1016/s0006-8993(96)01468-0

[45] Isomura R, Kitajima K, Sato C (2011) Structural and functional impairments of polysialic acid by a mutated polysialyltransferase found in schizophrenia. J Biol Chem 286(24):21535–45. 10.1074/jbc.M111.221143 21464126 PMC3122212

[46] Kanato Y, Kitajima K, Sato C (2008) Direct binding of polysialic acid to a brain-derived neurotrophic factor depends on the degree of polymerization. Glycobiology 18(12):1044–53. 10.1093/glycob/cwn084 18796648

[47] Seki T, Arai Y (1999) Different polysialic acid-neural cell adhesion molecule expression patterns in distinct types of mossy fiber boutons in the adult hippocampus. J Comp Neurol 410(1):115–25. 10397399 10.1002/(sici)1096-9861(19990719)410:1<115::aid-cne10>3.0.co;2-c

